# Agnew’s Surgery

**Published:** 1884-08

**Authors:** 


DR. AGNEW'S SURGERY.—A notice of the third volume of Dr. Haynes Agnew's great work, The Principles and Practice of Surgery, received from the publishing company of J. B. Lippincott, Philadelphia, should have been acknowledged by a befitting notice in the *Register*. The then editor, Dr. J. H. Logan, now hastens to make the amende honorable in the pages of the Atlanta Medical and Surgical Journal, both for this and Dr. Bartholow's valuable work on Materia Medica and Therapeutics.

The completion of the third volume terminates the labors of Dr. Agnew on this, perhaps, the greatest enterprise of his busy life. It comes in full time to supply a want beginning to be widely felt in this country—a complete practical treatise in the department of Surgery. In no field of the profession has so great advancement been made in the last two decades as in this. It would be interesting, in the proper place, to inquire into the causes that have given this prominence to surgery. Does it come from the experience and teachings of the recent great wars in America, the East and Continental Europe? Or is it the outcome of the genius and thought of the remarkable assembly of great men who, in the last quarter of a century, have illustrated the profession of medicine and surgery in both hemispheres?

Let the answer be as it may, it is certain that Dr. Agnew has succeeded in embodying, and publishing for the use of his brethren, a work which is at once a monument of industry, scientific learning and practical methods. It is not exhaustive, it is true, on any of the numerous subjects it presents, but it is something far better, a work of condensed, practical thought, to which the busy practitioner may hurriedly resort on the spur of the moment, and be sure of finding just what he most needs.

The third volume alone contains five hundred and fourteen illustrations, and most of them entirely new and original.

We received at the same time, from D. Appleton & Co., New York, a Practical Treatise on Materia Medica and Therapeutics, by Roberts Bartholow, A.M., M.D., LL.D, the fifth edition, revised and enlarged. Some sharp criticism has appeared on this work, but it will continue to hold its place as one of the best, most elaborate treatises we possess in the intricate field which it essays to cultivate. As a ready source of reference for the physiological and therapeutical action of drugs and curative methods, it has no rival in medical literature.

